# Sputter Deposited Magnetostrictive Layers for SAW Magnetic Field Sensors

**DOI:** 10.3390/s21248386

**Published:** 2021-12-15

**Authors:** Lars Thormählen, Dennis Seidler, Viktor Schell, Frans Munnik, Jeffrey McCord, Dirk Meyners

**Affiliations:** 1 Institute for Materials Science, Kiel University, Kaiserstraße 2, 24143 Kiel, Germany; dese@tf.uni-kiel.de (D.S.); visc@tf.uni-kiel.de (V.S.); jmc@tf.uni-kiel.de (J.M.); dm@tf.uni-kiel.de (D.M.); 2 Institute of Ion Beam Physics and Materials Research, Helmholtz-Zentrum Dresden-Rossendorf (HZDR), 01328 Dresden, Germany; f.munnik@hzdr.de

**Keywords:** magnetron sputter deposition, FeCoSiB, ERDA, XRD, film stress, magnetic field sensor, magnetic properties, magnetic domains, SAW

## Abstract

For the best possible limit of detection of any thin film-based magnetic field sensor, the functional magnetic film properties are an essential parameter. For sensors based on magnetostrictive layers, the chemical composition, morphology and intrinsic stresses of the layer have to be controlled during film deposition to further control magnetic influences such as crystallographic effects, pinning effects and stress anisotropies. For the application in magnetic surface acoustic wave sensors, the magnetostrictive layers are deposited on rotated piezoelectric single crystal substrates. The thermomechanical properties of quartz can lead to undesirable layer stresses and associated magnetic anisotropies if the temperature increases during deposition. With this in mind, we compare amorphous, magnetostrictive FeCoSiB films prepared by RF and DC magnetron sputter deposition. The chemical, structural and magnetic properties determined by elastic recoil detection, X-ray diffraction, and magneto-optical magnetometry and magnetic domain analysis are correlated with the resulting surface acoustic wave sensor properties such as phase noise level and limit of detection. To confirm the material properties, SAW sensors with magnetostrictive layers deposited with RF and DC deposition have been prepared and characterized, showing comparable detection limits below 200 pT/Hz^1/2^ at 10 Hz. The main benefit of the DC deposition is achieving higher deposition rates while maintaining similar low substrate temperatures.

## 1. Introduction

Surface acoustic wave (SAW) devices constitute a multifunctional sensor concept [1]. The use of different piezoelectric substrates and crystallographic orientations allows the excitation of various types of surface acoustic waves, like seismic waves such as Rayleigh waves or shear horizontal waves called Love waves. Depending on the different surface waveforms, sensors can be used for different measurement applications such as biological molecule detection [2,3] and temperature [4]. A large area of application for SAW sensors is a magnetic field sensor [4,5,6,7,8] and a tabular comparison can be found in the Appendix A (see Table A1). The sensors are based on various substrates and magnetic sensitive layers. Typical substrates are St-cut Quartz in different cutting directions, LiNbO_3_ and LiTaO_3_ [2,3,4,5,6,7,8,9,10,11,12]. In that context, a wide range of ferromagnetic materials are used for the magnetic sensitive layer, e.g., FeCo, FeGa, Fe_2_Tb and (Fe_90_Co_10_)_78_Si_12_B_10_ (FeCoSiB) [10,11,12], which are deposited on the SAW sensor as full films, multilayers or patterned structures [12,13,14]. The variety of magnetic layers display different magnetic behavior, which is strongly correlated to the magnetic anisotropies of the magnetic films. Controlling and adapting these for the respective layer system and utilizing them is one of the major challenges in the development of these magnetic systems for SAW devices. The shown research is based on ST-cut quartz SAW devices with a full film FeCoSiB layer based on previous studies [5,8,9] with a focus on the deposition process of the magnetic sensitive layer with regard to material composition, magnetic anisotropy, film stress, amorphicity and overall sensor performance.

In general, the working principle of SAW sensors is the generation of elastic waves based on the inverse piezoelectric effect by applying a high frequency voltage to interdigital transducers (IDT) deposited on a piezoelectric substrate.

As mentioned before, the Love wave sensors utilize horizontal shear waves. The waves propagate along a delay line while the wave energy is concentrated at the sensor’s surface by a guiding layer. To allow external influences to affect the sensor, it can be equipped with an additional functional layer on the delay line. For the purpose of magnetic field sensing, this functional layer consists of a magnetostrictive material. External magnetic fields cause the magnetostrictive material to change the effective shear and young’s modulus, affecting the velocity of transmitted Love waves in the guiding layer. Using opposite output IDTs, the voltage generated in the piezoelectric material by the transmitted and influenced shear wave is recorded and the phase change between input and output signal serves as a measure of the applied magnetic field. In this case, a magnetostrictive layer was deposited from a magnetron sputter target with the composition (Fe_90_Co_10_)_78_Si_12_B_10_ (FeCoSiB) on top of the delay line. The general characteristics of the used sensor concept are shown in ref Kittmann et al. [5].

In previous studies, it was shown that the preparation of a magnetostrictive layer presents a particular challenge in terms of control of magnetic anisotropy and magnetic domain structure [8]. Although methods exist to subsequently adjust the magnetic anisotropy of the film by means of heat treatments, these cannot be directly transferred to quartz substrates. Unlike silicon (100)-based magnetic field sensors, quartz substrates for SAW applications exhibit anisotropic thermal expansion, resulting in uniaxial film stress in the magnetostrictive layer with thermal load. As a result, these (uniaxial) film stresses contribute directly to the effective magnetic anisotropy in these films [15]. To minimize the effects of film stress, changes due to anisotropic thermal expansion, deposition processes have to be adapted to quartz to reduce the substrate temperature during deposition. On the other hand, film stress generated by the deposition can be beneficially utilized to influence the magnetic properties of the magnetostrictive film. By introducing an additional stress anisotropy to the induced magnetic anisotropy, it is possible to imprint an improved alignment of the magnetic anisotropy and, thus, the magnetic domain structure in soft magnetic material [16].

One of the most commonly used magnetron sputtering methods is based on radio frequency (RF) sputter operation which has the advantage that thicker magnetic target materials with strong magnetron fields can be used. For this reason, it is possible to use the powder-sintered material FeCoSiB, as the manufacturing process and machining of the target material requires a base thickness of several millimeters. However, the method has a high energy input on the substrate and the resulting deposition rates are low. To compensate for exhibited substrate heating, complex and expensive methods for sample cooling must be applied. As an alternative, cooling phases with plasma interruption were added to the deposition process [8,9].

This publication investigates the application of direct current (DC) deposition for magnetostrictive layer preparation on quartz substrates with the aim of reducing the energy input into the substrate and increasing the deposition rate. To enable DC operation, a magnetically adapted FeCoSiB target is used. An important aspect is the chemical composition of the resulting thin films. The soft-magnetic behavior of FeCoSiB depends on the concentration of silicon and boron atoms, which serve as glass formers between iron and cobalt [17]. The use of these elements makes the sputter-deposited layers amorphous and by this eliminates magneto-crystalline anisotropy effects on the magnetic properties. It must therefore be ensured that despite the change of deposition mode, the composition of the films is unaffected so that the film structure remains amorphous. These amorphous magnetic films show good soft-magnetic properties [18] together with a high magnetostrictive response, as desired for use in SAW-based magnetic field sensors. It will be shown that a changeover to DC deposition enables significantly higher deposition rates without a significant alteration in structural and magnetic properties, as well as in SAW sensor performance. For this purpose, the chemical, structural and magnetic properties of DC and RF with different sputter pressure-deposited FeCoSiB films are investigated. The corresponding SAW sensor performance with RF and DC deposited magnetic films is compared in terms of sensitivity and noise.

## 2. Materials and Methods

The required magnetostrictive FeCoSiB layers for the experiments are deposited in a VonArdenne CS730S sputtering system. The system has nine planar magnetron sputter sources and allows the deposition of materials as adhesion or passivation layers without breaking the vacuum. FeCoSiB depositions are performed from a multi-element target with 99.95% purity and at constant power for RF and DC of 200 W with argon gas flow of 40 sccm. The chamber pressure is varied for the depositions in the range of 1.0 × 10^−3^ mbar to 1.0 × 10^−2^ mbar with a step size of 1.0 × 10^−3^ mbar. The depositions cannot be performed in one cycle. Deposition time limits are necessary to keep the energy input into the sample and the resulting sample temperature low to minimize thermal expansion-induced film stress. Depending on the chamber pressures, deposition times are adjusted to deposit 5 nm FeCoSiB by RF mode and 20 nm FeCoSiB by DC mode. As a result, for the preparation of in total 200 nm thick FeCoSiB layers, 40 cycles and 10 cycles are run for RF and DC, respectively. To ensure that the maximum temperature of the substrate does not exceed 30 °C, cooling breaks of 300 s (RF) and 180 s (DC) are introduced between deposition cycles. As an additional measure, 2 mm thick AlO_2_ spacers are placed below the samples to thermally decouple the SAW substrate from the aluminum sample carrier.

**Figure 1:**
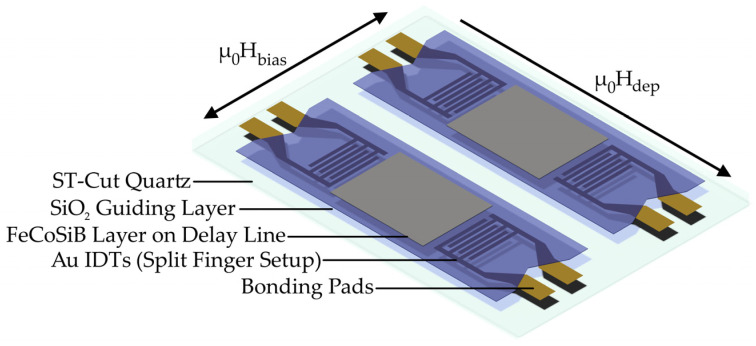
Schematic drawing of a SAW chip in use with two SAW delay line sensors, each with an independent guide layer. For magnetic anisotropy imprinting a magnetic field µ_0_H_dep_ is used during deposition of the magnetic layer FeCoSiB. The sensors are operated with µ_0_H_bias_ at the magnetic operating point during sensor operation.

For the fabrication of FeCoSiB layers on SAW substrates with a thickness of 200 nm, the Ar pressure is chosen to achieve a compressive film stress of 200 MPa. Thus, for an Ar pressure of 2.5 × 10^−3^ mbar, the rate for RF is 0.16 nm/s with a cycle sequence of 30 s deposition time and 300 s deposition pause. For DC, on the other hand, with a chamber pressure of 2.0 × 10^−3^ mbar, the rate is 0.4 nm/s with a cycle sequence of 50 s deposition/180 s cooling. As a result, the total processing time is reduced from approx. 4.5 h for RF to 1.5 h for DC for the aimed FeCoSiB thickness. A uniaxial magnetic anisotropy is induced in the films parallel to the SAW propagation direction by applying a static permanent magnetic field of µ_0_H_dep_ = 70 mT during film deposition (see Figure 1). This field is generated by two parallel aligned Nd_2_Fe_14_B permanent magnets with dimensions of 25 mm × 6 mm × 2 mm. The magnets are connected by an iron yoke to increase the strength and homogeneity of the generated magnetic field. The magnet is used for all depositions.

The deposited FeCoSiB films are chemically characterized by elastic recoil detection analysis (ERDA) [19], which enables the detection of light elements in a heavy element matrix. For the chemical analysis, 10 mm × 10 mm silicon substrates (100) with native oxide are used. These substrates are coated with a stack of 5 nm Cr/400 nm FeCoSiB/10 nm Ta. Here, chromium serves as an adhesion promoter. Tantalum is used for surface passivation to prevent early corrosion of the FeCoSiB. The ERDA measurements are performed with a Cl^7+^ ion beam and two different energies of 43 MeV and 35 MeV. The angle of incidence on the film is 75° with respect to the normal sample. The irradiated film area has a size of 2 mm × 2 mm. The scattered Cl ions and recoiled target atoms are detected at an angle of 30° with a Bragg ionization chamber (BIC), which allows the energy and the atomic number to be determined. The last is necessary to identify the particle. To measure the total energy, the particles must be stopped completely in the BIC, which is filled with isobutane at a pressure of 125 mbar. This pressure is optimal for the measurement of recoil atoms of C to P and scattered Cl ions produced by a beam energy of 43 MeV. Because the recoil B atoms are light and have a long-range, a separate measurement with a beam energy of 35 MeV has been performed, which is sufficiently low for the B atoms to be completely stopped in the detector. The output of the BIC detector is a 2D-histogram where the number of particles is displayed as a function of energy and Bragg peak signal [20]. In this 2D plot, the elements can be identified in branches, detaching from the main branch of scattered Cl ions. The analysis depth for the recoil atoms is based on where the recoil branch detaches from the main branch and it is highest for atoms with low atomic number (Z). For Si, the analysis depth is relatively low as can be observed in the depth profiles (Section 3.1). H recoil atoms have been detected with a separate solid-state detector at a scattering angle of 40°. This detector is preceded by a 25 µm Kapton foil to stop scattered ions and heavy recoil ions. The depth resolution of this system is reduced because of energy loss straggling in the foil. A separate detector is needed because the range of the H atoms is too large to stop in the BIC detector. The processing of the measured spectra and calculation of elemental depth profiles is performed using NDF v9.3g [21].

As described, B and Si serve as glass formers and prevent the formation of crystalline iron-cobalt phases. Provided that enough of the glass formers is incorporated, an amorphous film structure is expected to form during sputter deposition. To investigate the structure by X-ray diffraction (XRD) method, samples are prepared from silicon (100) substrates with native surface oxide. The sample size is 15 mm × 15 mm to avoid beam overspill. The substrates are coated by 10 nm Ta/200 nm FeCoSiB. These layers are patterned by lithography and lift-off process to create a circular FeCoSiB layer with an area of 150 mm². No passivation layer is used for the XRD measurements, which are executed timely after layer deposition. To examine for the X-ray amorphous state, a Rigaku Smartlab 9 kW XRD tool is operated in grazing incidence (GIXRD) geometry. Generating Cu Kα_1_ and Kα_2_ radiation, the source is run at a power setting of 200 mA and 45 kV. The resulting diffraction patterns are recorded with a 2D detector.

Another important aspect when considering the magnetic performance on the SAW sensors is the film stress of the magnetostrictive layer. For the determination of the respective film stress, cantilevers made of “UPILEX-S^®^” in a size of 25 mm × 2.5 mm × 50 µm are used. The cantilevers are coated with a 10 nm layer of Ta before magnetic coating and reference measurement, to improve adhesion and reflection properties. The bending of the cantilevers before and after deposition of 200 nm FeCoSiB at varying chamber pressures is recorded and evaluated using a Keyence VK100 laser confocal microscope. The Stoney equation [22] is used to calculate the resulting film stresses. In order to investigate the influence of film stress on the imprinting of magnetic anisotropy by the external magnetic field, additional magnetic analysis samples based on 10 mm × 10 mm silicon (100) substrates with native oxide are prepared for magnetic property investigations. These samples are coated with the layer system 10 nm Ta/200 nm FeCoSiB/10 nm Ta. In this case, the Ta layers act as adhesion promoter and passivation layer, respectively. The deposition of FeCoSiB for the RF and DC samples is performed at the deposition pressures between 2 × 10^−3^ mbar and 1 × 10^−2^ mbar.

**Figure 2:**
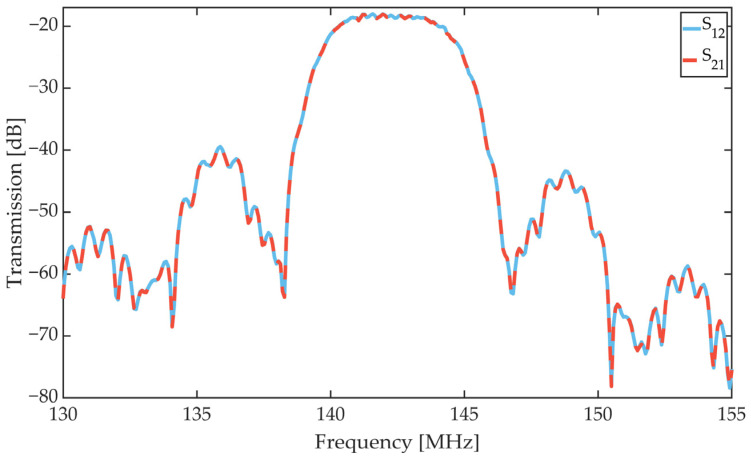
Scattering parameters (S12 and S21) of an SAW sensor used in this publication measured in magnetic saturation. The center frequency of the Love wave device is at 142.5 MHz.

The SAW sensors used for this work are based on the above-mentioned ST-Cut quartz and have two SAW sensors with independent delay line on a sensor chip of size 14 mm × 8.9 mm. The single sensors have two pairs of split finger IDTs with a periodicity of 28 µm and Figure 2 shows the scattering parameters (S12 and S21) for a sensor used in this publication. The center frequency is at 142.5 MHz. All sensors investigated show similar scattering parameters in the range between 141 MHz and 146 MHz. The variance of the center frequency is due to thickness differences in the SiO_2_ wave guiding layer.

The design and fabrication of the SAW sensors has been described in detail in publications [5,8]. To evaluate the sensor properties, the SAW sensors are mounted on printed circuit boards (PCB) and the impedance of input and output is matched to 50 ohm. The measurements are performed using a Zurich Instruments UHFLI lock-in amplifier, a vector network analyzer and a Rohde and Schwarz FSWP phase noise analyzer in a shielded chamber [23,24]. Scattering parameters, magnetic sensitivity and phase noise are measured. A more detailed description of the measurement procedure can be found in Schell et al. [8]. The cross-sensitivity to temperature of SAW sensors is well known and can be mitigated by using a second SAW device without magnetostrictive coating for compensation [25,26]. In the experiments presented here with a focus on the influence of the magnetic layer, the temperature in the lab environment was kept constant and a compensation method was not applied. An additional approach to reduce these influences on ST-Cut quartz resonators is shown in Mishra et al. [27].

For a better understanding of the magnetic layer behavior of the fabricated SAW magnetic field sensors, large view magneto-optical Kerr effect (MOKE) microscopy investigations are performed [28]. The large view microscope has the ability to image the entire delay line and capture the magnetization loops and magnetic domain images, also on the device level. For this purpose, the above-mentioned magnetic analysis samples, as well as the magnetic SAW sensors, were considered. Magnetic domain images are taken in the demagnetized state and around the devices’ working points to analyze the orientation of the magnetic domains and their overall domain structure. To investigate the magnetic behavior in more detail, longitudinal and transversal magnetization loops of the device samples were measured with the application of magnetic field parallel and perpendicular to the imprinting field µ_0_H_dep_ in the MOKE microscope. The magnetic hysteresis losses are extracted from the magnetization loops obtained for films with different conditions of film depositions.

## 3. Results

### 3.1. Chemical and Structural Analysis

**Figure 3:**
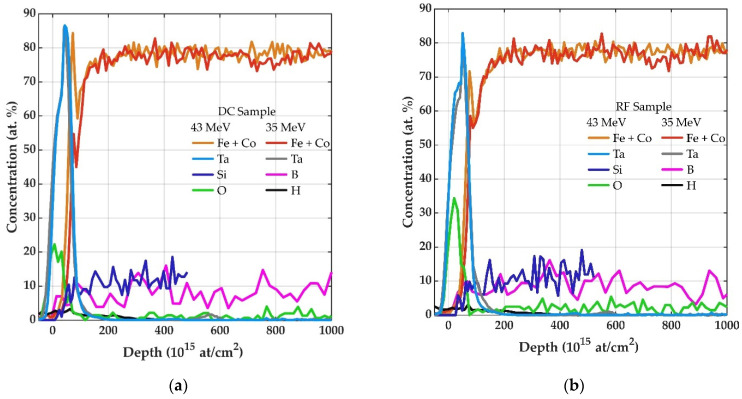
(**a**,**b**) ERDA depth profile of FeCoSiB deposited with DC and RF. The samples consist of a 5 nm Cr/400 nm FeCoSiB/10 nm Ta layer. The tantalum layer serves as a protection layer to prevent oxidation. The displayed measurement depth range corresponds to approximately 120 nm. As indicated, two different accelerating voltages 43 MeV and 35 MeV were used to obtain optimal results for all elements.

The ERDA depth profiles of DC (a) and RF (b) deposited FeCoSiB layers in Figure 3 show the elemental distributions in at.% as a function of profile depth in at/cm^2^. Based on the estimated densities of the measured elements, an overall measurement depth of 420 nm (equal to 3526 at/cm^2^) can be assumed. In Figure 3, the depth profiles recorded at accelerator energies 43 MeV and 35 MeV for a depth of 120 nm are merged for the measured elements iron (Fe), cobalt (Co), silicon (Si), oxygen (O), carbon (C), boron (B) and hydrogen (H). 

The concentration data are derived from the measured ERDA and Cl scattering spectra and have been calculated using NDF v9.3g. A special case arises for the elements Fe and Co. Due to their similar masses, Fe and Co signals cannot be differentiated from each other and are therefore summed. The uncertainty of the determined elemental concentration of the performed measurement is in the 1 at % range for the elements (Fe+Co), Si and B. Due to the low amounts of oxygen, carbon and hydrogen (close to the detection limit) the relative error of O content is 10% and 20–30% for C and H. Furthermore, the depth profiles are convoluted by the system resolution, physical effects such as straggling and isotope distribution for B, and also by the statistical fluctuations of the spectra [19]. 

From the elemental depth profiles, it can be concluded that the 10 nm thick Ta top layer has sufficient oxidation resistance and can prevent deep penetration of O. The concentration of O drops sharply with penetration depth, falling within the Ta layer to a low amount of about 2 at.%, which remains constant till the end of the depth profile. Apparently, O is incorporated into the film during deposition. Possible oxygen sources include chamber leakage, sputter gas impurities, and the use of powder-sintered target material. The last one is assumed to be the most likely source due to the manufacturing method of powder-sintered targets. Furthermore, it can be seen in Figure 3 that in both deposition cases the element distribution after the Ta top layer is constant. This provides information that no measurable compositional variation is formed by the pause times between the deposition cycles for DC and RF and the FeCoSiB grows as a continuous film. The origin of the elements C and H are assumed to be due to a residual gas content in the deposition chamber. Furthermore, manufacturing and processing of the FeCoSiB target for the magnetic adaption could cause the incorporation of the detected C impurities.

However, it should be noted that the configuration of the ERDA setup with BIC was chosen to enable the detection of the target elements, whereas the Ar detection requires a configurational change. To improve the mass separation between Ar atoms and Cl ions, ERDA can be used with a time-of-flight analysis system (ToF). Additional ToF-ERDA experiments reveal an upper limit of 0.5 at.% for the Ar concentration in the FeCoSiB layers. The elemental concentrations averaged across the measured FeCoSiB cross-sections are summarized in Table 1.

Fe and Co together reach a proportion of about 78 at.%, which equals the content in the sputter target with the nominal composition of (Fe_90_Co_10_)_78_Si_12_B_10_. Si also reaches the target value of 12 at.%. B, on the other hand, deviates from the desired concentration of 10 at.% by more than 1 at.%. Within the mentioned measurement error, the ERDA measurements reveal the same elemental concentrations for both deposition methods RF and DC.

**Figure 4:**
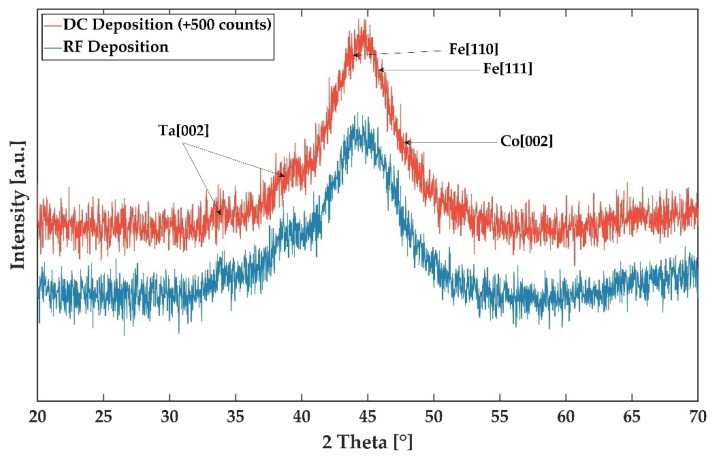
XRD result of Ta/FeCoSiB thin films deposited by RF and DC mode at 2.5 × 10^−3^ mbar and 2.0 × 10^−3^ mbar, respectively. For both deposition processes, the power was set to 200 W at an Ar flow of 40 sccm. Grazing incidence XRD was used with 2 Theta ranging from 20° to 70° with steps of 0.25°. For a better representation, an offset for the XRD result for the thin film deposited by DC mode was used. The adhesion promoter tantalum and a signal combination of the amorphous FeCoSiB are noticeable.

The structural analysis was performed by XRD as described above. Figure 4 shows the measured X-ray intensities of the two FeCoSiB films deposited by RF (DC) mode at a pressure of 2.5 × 10^−3^ mbar (2.0 × 10^−3^ mbar). The XRD spectra show two broad and smaller diffractions at 2 Theta equal to 34° and 39°, which originate from the buried Ta layer. The Ta adhesion promoter is still measurable due to the GIXRD penetration depth of more than 200 nm. Due to the low intensity, it is not possible to determine any precise information about the Ta structure. We assume a nanocrystalline or an amorphous structure. An amorphous structure was reported for thin tantalum layers prepared by magnetron sputter deposition [29]. The third reflex, and largest, extends around 44°. In this 2 Theta range, diffraction peaks can emerge from Fe at 44.77°, Co at 47.69° and FeCo at 44.97°. It is assumed that the main source of the signal is the high amount of Fe in the film. Broad and low-intensity distributions are measured in this 2 Theta range for possible Fe and Co signals. No further peaks are exhibited. Based on the results of the GIXRD, it can be assumed the amorphous FeCoSiB quality is the same, regardless of RF or DC deposition mode.

### 3.2. FeCoSiB Film Stress and Magnetic Behavior

**Figure 5:**
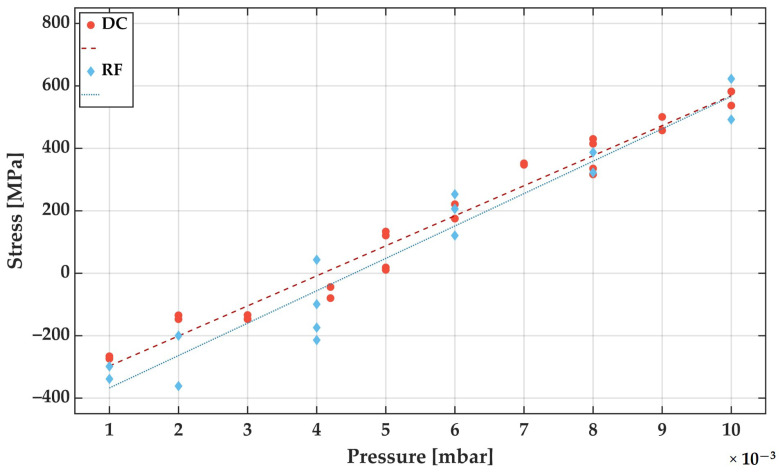
Determined film stress measurements for depositions performed with DC (red) and RF (blue) with different Ar deposition pressures. The resulting FeCoSiB film thickness was set to 200 nm and the initial and resulting bending is measured with a laser confocal microscope. The film stresses are calculated by the Stoney equation from the curvature of the cantilevers before and after film deposition. (The data are fitted with a simple linear regression).

After it has been demonstrated that both deposition methods DC and RF lead to the same layer composition and structure, the control of film stress and imprinted magnetic anisotropy become important parameters for the following magnetic samples and SAW sensors. The film stresses determined for this purpose are shown in Figure 5 as a function of Ar deposition pressure for RF and DC sputter deposition conditions.

It is clearly visible that both deposition modes show a similar film stress to deposition pressure relation. The overall course of the stress vs. Ar deposition pressure curves can be explained by the pressure-dependent mean free path of the film formers. At lower pressure the sputtered species retain their kinetic energy and a more compact film growth [30]. According to the assumed linear regression, the transition from compressive to tensile stress appears at around 4.0 × 10^−3^ mbar and 4.5 × 10^−3^ mbar for DC and RF deposition, respectively. For both deposition modes, the Ar deposition pressure can be adjusted to obtain FeCoSiB layers with low-stress magnitudes.

To relate the changes in film stress to the magnetic properties of the film, 10 mm × 10 mm sized samples with a 200 nm thick FeCoSiB layer were analyzed utilizing MOKE magnetometry and microscopy. For the measurement of the magnetization loops the region of interest was positioned in the center of the sample with a size of 5 mm × 5 mm, which approximately corresponds to the SAW sensor magnetic film size and position. Concentrating on the center of the films minimizes edge effects in the measurements of the magnetization reversal and thereby reflects the actual magnetic film properties.

**Figure 6:**
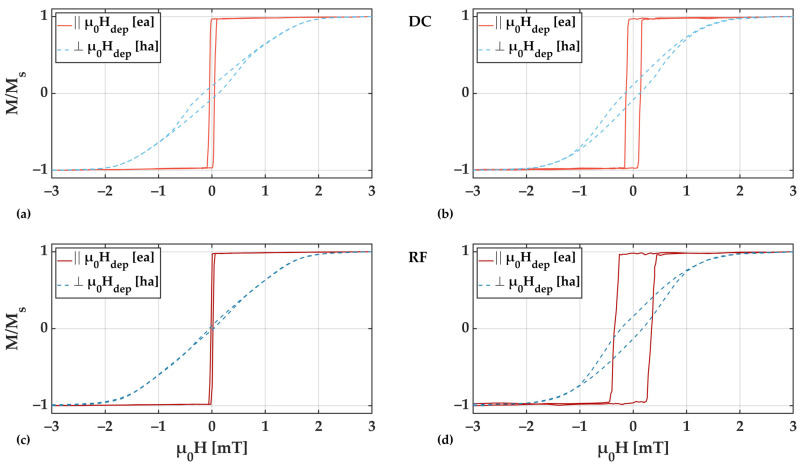
Magnetization curves for 200 nm thick FeCoSiB films deposited with DC (**a**,**b**) and RF (**c**,**d**) at an Ar deposition pressure of 2.0 × 10^−3^ mbar (**a**,**c**) and 8.0 × 10^−3^ mbar (**b**,**d**) measured along and perpendicular to µ_0_H_dep_, respectively along the presumed easy axis (ea) and hard axis (ha) of magnetization. The measured area of 5 mm × 5 mm was set to the sample center to avoid edge effects. The measurement field was applied parallel (red) and perpendicular (blue) to µ_0_H_dep_.

Magnetization loops obtained field from RF and DC deposited samples at deposition pressures of 2.0 × 10^−3^ mbar and 8.0 × 10^−3^ mbar are shown in Figure 6. The corresponding film stress is ≥200 MPa compressive and ≥300 MPa tensile for 2.0 × 10^−3^ mbar and 8.0 × 10^−3^ mbar, respectively. The magnetic field was applied from −3 mT to +3 mT parallel and perpendicular to the magnetic deposition field µ_0_H_dep_.

From the magnetization loops, clear uniaxial anisotropy characteristics can be derived with the easy axis (ea) of magnetization aligned along the direction of µ_0_H_dep_. In all cases, the coercivity of the magnetic films increases with tensile film stress.

**Figure 7:**
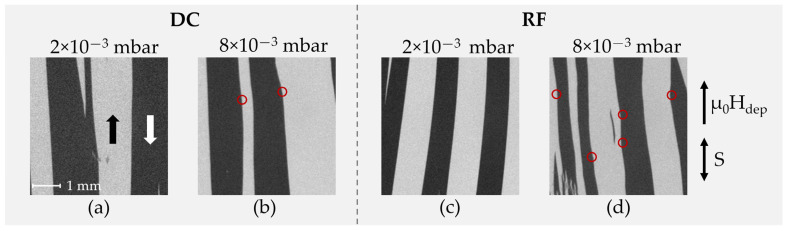
MOKE microscopy images of demagnetized magnetic samples deposited with 10 nm Ta/200 nm FeCoSiB /10 nm Ta with an applied magnetic deposition field µ_0_H_dep_ during deposition, at Ar deposition pressures of 2.0 × 10^−3^ mbar (**a**,**c**) and 8.0 × 10^−3^ mbar (**b**,**d**) for DC (**a**,**b**) and RF (**c**,**d**) sputter-deposited samples. The magneto-optical sensitivity S is aligned parallel to the µ_0_H_dep_. The FeCoSiB films with low deposition pressure and high compressive film stresses (**a**,**c**) show a slight misalignment of the magnetic domain walls by up to 5° as compared to the direction of µ_0_H_dep_. At high deposition pressures and high tensile stresses (**b**,**d**), magnetic domain wall pinning centers (red circles) appear in the magnetic layer.

In Figure 7, corresponding magnetic domain images obtained at zero field from the same RF and DC deposited samples are shown. Small tilts (up to 5°) of the induced anisotropy axis are visible as a result from slight field inhomogeneities in the deposition field setup (µ_0_H_dep_) and/or deviations in the position in the sputtering chamber.

For both deposition pressures the formation of large and well-aligned magnetic domains becomes visible. In contrast to the 2.0 × 10^−3^ mbar samples, the 8.0 × 10^−3^ mbar samples show areas of local domain wall pinning (red circles in Figure 7), which are particularly evident for the case of RF deposition. This is an indication of magnetically active defects, which are related to magnetic property inhomogeneities. The domain wall pinning becomes also visible during the magnetization reversal along the magnetic easy axis (not shown). It is directly reflected in the variations seen in the magnetization loops (Figure 6). Considering the high tensile stresses, the presumed magnetic property variations are assumed to be due to local magneto–elastic interactions, which directly influence the magnetic behavior [31]. The exact nature of the defects cannot be concluded from our measurements.

Overall, comparing the measured magnetization loops at low and high deposition pressure, a significant broadening is visible for higher pressures, which correlates directly with the observed changes in magnetic domain characteristics and substantiates the domain wall pinning by local stress fields due to an increase in stress magnitude. 

**Figure 8:**
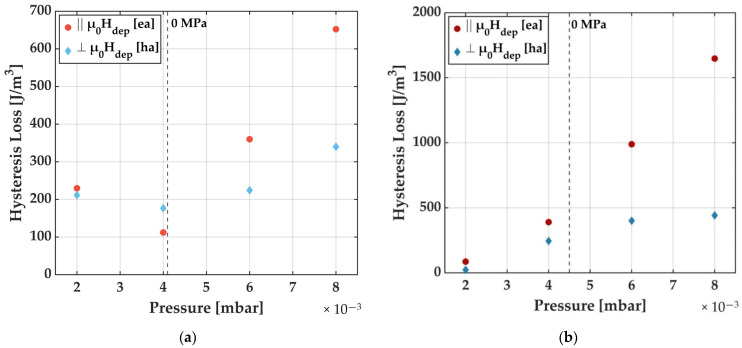
Hysteresis losses for DC (**a**) and RF (**b**) deposited FeCoSiB films as a function of Ar deposition pressure. The values in (**a**,**b**) were obtained from hysteresis curves of magnetic test samples with a measured area of 5 mm × 5 mm located in the center of the samples. To determine the hysteretic losses, the enclosed area of the hysteresis curves was determined according to the orientation parallel and perpendicular to µ_0_H_dep_. The approximate positions of zero stresses are indicated.

An accurate parameter to quantify the change in the magnetization loops is the hysteresis loss. The hysteresis loss is especially important as it is strongly connected to the exhibited magnetic noise in magnetoelectric composite magnetic field sensors [32,33,34]. Data for the whole parameter range are displayed in Figure 8a for DC and Figure 8b for RF deposition for Ar deposition pressures ranging from 2.0 × 10^−3^ mbar to 8.0 × 10^−3^ mbar. A saturation magnetization of M_s_ = 1193 kA/m [35] was assumed for the extraction from the MOKE loops. 

For both deposition methods, a trend to higher hysteresis losses with higher Ar deposition pressure is visible. Although we assume that the FeCoSiB films deposited with RF and DC have the same chemical composition (ERDA) and structure (XRD), we see a clear trend in the change of magnitude of magnetic losses. From the magnetic domain studies, we relate this to inhomogeneities in the magnetic films that, in connection with the magnetostrictive material, hinder the magnetization process. Magnetic domain wall pinning and a reduction of small field permeability arise from the magnetoelastic interactions. It is well known that CoFe-based amorphous soft-magnetic alloys display a minimum in coercivity with zero magnetostriction [36] due to the exhibited coupling between stresses and magnetic properties.

### 3.3. SAW Sensor Performance

As shown in the previous chapter, a uniaxial anisotropy cannot be induced precisely with increasing sputtering pressure. Therefore, the FeCoSiB films on the SAW devices are deposited at sputtering pressures, which correspond to film stresses of −200 MPa (compressive stress), i.e., 2.0 × 10^−3^ mbar for DC deposition and 2.5 × 10^−3^ mbar for RF deposition (see Figure 5).

**Figure 9:**
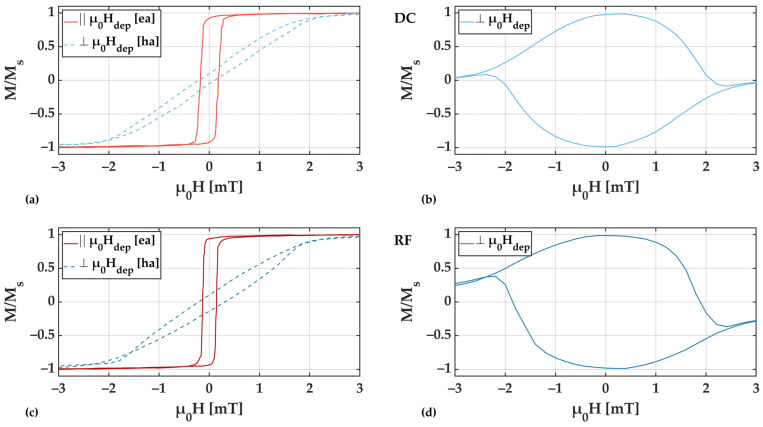
Longitudinal and transversal magnetization curves for a 200 nm thick FeCoSiB film deposited with DC sputter deposition at an Ar pressure of 2.0 × 10^−3^ mbar (**a**,**b**) and with RF sputter deposition at an Ar pressure of 2.5 × 10^−3^ mbar (**c**,**d**) from SAW devices. Longitudinal curves were measured along (red) and perpendicular (blue) to µ_0_H_dep_, the latter being perpendicular to the SAW propagation direction. The measured area was set in accordance with the active SAW device area.

An analogous magnetic analysis as shown before, including magnetization loop measurements and magnetic domain analysis, was performed directly on the SAW devices. The magnetic analysis was further restricted to the active region of the SAW device. Magnetization loops with the magnetic field along and perpendicular to the direction of wave propagation are shown in Figure 9. The magnetic field direction perpendicular to the deposition field (⊥ µ_0_H_dep_) corresponds to the applied bias field in SAW sensor operation defining the working point. To get a clearer view on the overall magnetization reversal behavior also the transverse magnetization loops [32,37] are analyzed.

**Figure 10:**
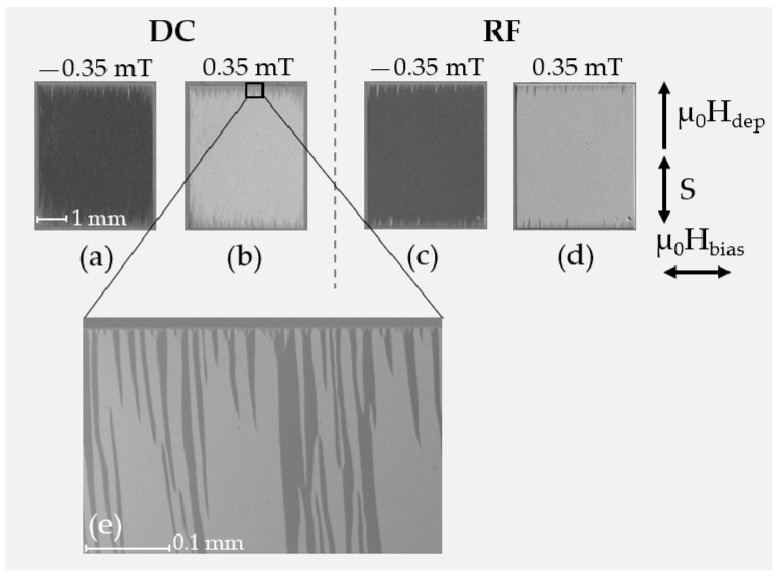
MOKE images from DC (**a**,**b**,**e**) and RF (**c**,**d**) sputter deposited SAW structures at magnetic bias fields µ_0_H_bias_ of −0.35 mT and +0.35 mT aligned perpendicular to µ_0_H_dep_ (as indicated). The magneto-optical sensitivity (S) is aligned parallel to µ_0_H_dep_. Nearly single magnetic domain behavior occurs. Yet, spike domains (**e**) [38] at the bottom and top edges (⊥ to µ_0_H_dep_)) form. Shown in (**e**) is a higher resolution (20×) version of image (**a**) in the area of the upper edge and shows the spike domains.

Corresponding magnetic domain images at magnetic bias fields µ_0_H_bias_ of −0.35 mT and +0.35 mT coming from negative and positive saturation, respectively, are displayed in Figure 10. 

The magnetic hysteresis effects are more pronounced as in the case of the extended thin films, which we attribute to magnetic domain effects at the sample edges. Spike domains extending in the active region of the sensor become visible in Figure 10e, which hinder the magnetization reversal and which lead to an increase in coercivity for the magnetic field directions along and perpendicular to µ_0_H_dep_. For the magnetic field direction perpendicular to µ_0_H_dep_ we obtain a coercivity field of µ_0_H_c_ ≈ 0.15 mT for RF and µ_0_H_c_ ≈ 0.3 mT in the case of DC sputter deposition. From the transverse magnetization loops (Figure 9b,d), a mostly coherent rotation of magnetization is obtained for the horizontally aligned magnetic bias fields. Despite the rotational process, unity of magnetization is not reached due to the spike domain formation at the top and bottom edge. The lower amplitude in the general magnetic and magnetic domain behavior of the magnetostrictive phase of the SAW sensors is comparable. The hysteretic magnetization reversal behavior has a direct influence on the SAW sensor characteristics.

**Figure 11:**
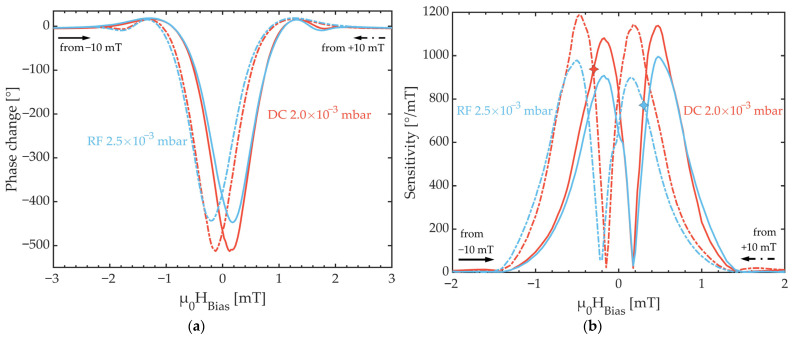
(**a**) Phase change of SAW sensors as a function of applied DC bias field for DC (red) and RF (blue) deposited FeCoSiB films with low compressive film stress. (**b**) Measured sensitivity as a function of applied DC bias field obtained using a 1 µT AC field for DC (red) and RF (blue) deposited FeCoSiB films with compressive film stress. Markings in the DC and RF curves highlight the magnetic bias field in which the best LOD measurement has been achieved. All curves in (**a**,**b**) were recorded from −10 mT to +10 mT and vice versa. The measurement step size in the range from −1 mT to +1 mT is 25 µT. AC and DC fields were applied perpendicular to the SAW propagation direction.

Figure 11a shows the phase change of SAW sensors with DC and RF deposited FeCoSiB films under the intrinsic compressive stress as a function of an applied DC bias field µ_0_H_Bias_. The field was changed from −10 mT to +10 mT and vice versa with a step size of 25 µT in the most relevant field range from −1 mT to +1 mT. 

With 510° for the DC sputtered sensor and 450° for RF the total phase changes are very similar for both deposition techniques. In fact, several sensors have been measured to confirm the obtained results and all show maximum phase changes between 430° and 510°. The hysteresis characteristics are in accordance with the magnetometric analysis of the obtained bias curves, i.e., the large hysteresis between the two field scanning directions for each deposition technique. While in an ideal hysteresis-free case both phase minima are expected to be at 0 mT bias field [5], they are around the coercive fields at −0.125 mT and +0.125 mT for the DC deposition case and −0.2 mT and +0.175 mT for the RF deposited films. Nevertheless, the bias curve hysteresis is very similar for both deposition techniques and therefore independent of the type of deposition bias.

In Figure 11b, the measured sensitivity, i.e., the derivative of the phase change, shows two characteristic sensitivity maxima for each measurement direction and each sensor. The maxima indicate the positions of the highest slope in the static bias curves in Figure 11a. These points of highest sensitivity for the RF deposition case are at −0.175 mT and +0.475 mT coming from negative saturation with maximum sensitivities of 907°/mT and 996°/mT, respectively, and at +0.15 mT and −0.5 mT coming from positive saturation with maximum sensitivities of 900°/mT and 978°/mT, respectively. For the DC deposited films, these respective points are at −0.175 mT and +0.475 mT coming from negative saturation and +0.175 mT and −0.475 mT coming from positive saturation with sensitivity of 1081°/mT, 1138°/mT, 1144°/mT and 1188°/mT, respectively. The slight differences in total phase change and sensitivities originate from slightly varying FeCoSiB thicknesses [9] and tilted orientation of magnetic anisotropy [8]. However, the values presented here are in good agreement with previously published results covering a sensitivity range from 500°/mT to 2000°/mT [5,8,9,32].

**Figure 12:**
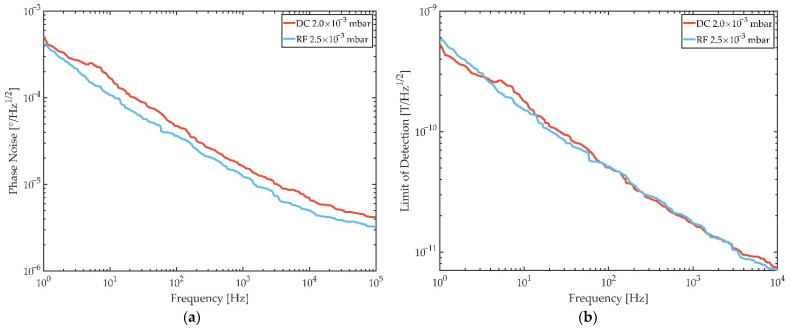
(**a**) Phase noise as a function of frequency from the SAW excitation carrier for DC (red) and RF (blue) deposited FeCoSiB films with compressive film stress. The shown phase noise spectra are measured at magnetic bias fields and excitation powers, which lead to the lowest limits of detection. In the DC deposition case this corresponds to a bias field of −0.3 mT (from negative saturation) and an excitation power of 1 dBm, and in the RF deposition case this is +0.35 mT (from positive saturation) and 2 dBm. (**b**) Limit of detection as a function of frequency from the SAW excitation carrier for DC (red) and RF (blue) deposited FeCoSiB films with compressive film stress. The limit of detection is the quotient of a sensors phase noise and its sensitivity.

While the sensitivity is a useful sensor characteristic, the measure which ultimately quantifies the performance of a magnetic field sensor, however, is the limit of detection (LOD). The LOD results from dividing the sensors noise, in this case phase noise, by its sensitivity. As shown in Figure 11b and Figure 12a, while the maximum sensitivity of the sensor with DC deposited FeCoSiB is higher than the phase noise is, in general, it is lower for the RF deposited films.

The phase noise spectra in Figure 12a are both recorded under sensor operating parameters, which lead to the lowest LOD for each sensor. More precisely, these parameters are the excitation power and µ_0_H_bias_. Phase noise spectra were measured for various DC bias fields and it turned out that the lowest LODs are achieved when applying a bias field slightly higher, i.e., slightly off the point of highest sensitivity. For the DC deposition case, this is at −0.3 mT coming from negative saturation and +0.35 mT coming from positive saturation for the RF case. For the excitation power, it was shown before that there exists an optimum excitation power where the phase noise is the lowest in SAW magnetic field sensors [32]. For the sensor design considered in Ref. [32] and here, this optimum excitation power is 1 dBm for the DC deposition case and for the RF case it is 2 dBm. Finally, this optimum operation leads to detection limits as low as 179 pT/Hz^1/2^ for DC deposited films and 152 pT/Hz^1/2^ for RF deposited films at 10 Hz, respectively. At 100 Hz the LODs are 51 pT/Hz^1/2^ (DC) and 52 pT/Hz^1/2^ (RF). 

However, it should be noted that the LODs of all sensors investigated for this study vary for both deposition types between 152 pT/Hz^1/2^ and 190 pT/Hz^1/2^ at 10 Hz and can therefore be considered equivalent. This shows that in terms of SAW magnetic field sensor performance with DC deposited films, the same LODs can be achieved while providing lower deposition times and less heat generation.

## 4. Conclusions

FeCoSiB thin films using DC magnetron sputtering for magnetic field SAW sensors were shown to give equivalent results in terms of magnetic properties as well as sensor performance as compared to commonly used RF deposited layers. 

Due to the higher deposition rate in DC operation, it becomes possible to reduce the total deposition time by a factor of 3, which is a significant improvement and advantage over the common RF sputter process. Furthermore, the chemical composition measured by ERDA and the structure measured by XRD of the prepared films are identical for DC and RF deposited FeCoSiB. Major factors influencing the magnetic performance of the SAW sensors such as film stress can be controlled and show that compressively stressed FeCoSiB films provide better control of the soft-magnetic film properties. On the SAW device level, it was shown that the magnetic layer properties and the resulting detection limits of the sensors with a single layer of 200 nm FeCoSiB are virtually the same with 51 pT/Hz^1/2^ (DC) and 52 pT/Hz^1/2^ (RF) at 100 Hz and with 179 pT/Hz^1/2^ on average at 10 Hz. Through further development of DC mode deposited magnetic films it will be possible to further improve the magnetic anisotropy of the thin film as well as to reduce the film variance. Thus, it will be possible to further improve the sensitivity, as well as the resulting LOD, with a significantly accelerated preparation process.

Another approach to improve the sensor performance is to use other structuring mechanisms, such as ion beam etching, to gain better control over the edge profile of the FeCoSiB, while also reducing possible defect structures caused by the resist needed for the lift-off process. Alternative magnetic layer systems, such as exchange bias stacks, show significant improvements in sensor performances [39,40,41]. However, due to the complexity of the exchange bias systems, the deposition times would increase significantly when using the RF mode, which can now be ideally compensated by DC film deposition.

## Figures and Tables

**Table 1:** Averaged elemental concentrations calculated from the FeCoSiB depth profile for an RF and DC sample deposited from a target material with a nominal composition of (Fe_90_Co_10_)_78_Si_12_B_10_.

Sample	Fe+Co (at.%)	Si (at.%)	O (at.%)	C (at.%)	B (at.%)	H (at.%)
RF	77.4	11.4	2.32	0.44	8.40	0.075
DC	78.2	12.0	1.63	0.52	7.63	0.056

## Data Availability

The data presented in this study are available on request from the corresponding author.

## Appendix A

**Table A1:** Chronological comparison of selected SAW magnetic field sensors from 2018 to 2021 based on substrate and magnetic layer.

Source	Substrate	IDT	SAW Guiding/Top Layer	Magnetic Layer	Wave Form	Operating Frequency	Sensitivity
This work	ST-cut quartz x + 90°	10 nm Ta/200 nm Au/10 nm Cr	4.5 µm SiO_2_	10 nm Ta/ 200 nm FeCoSiB/ 10 nm Ta	Love wave	144 MHz	1188°/m (2373 kHz/mT *)
Fahim 2021 [42]	ST-cut quartz (Murata RO3101)	Al	-	PVA + Ni/Fe nanopowder	-	433.92 MHz	5.83 kHz/mT
Mishra 2020 [43]	ST-cut quartz x + 90°	Al	400 nm ZnO	100 nm CoFeB	Love wave	421 MHz	0.75 kHz/mT
Yang 2020 [44]	ST-cut quartz x + 90°	100 nm Al	333 nm ZnO/ 400 nm SiO_2_	5 nm Ta/ 100 nm Co_40_Fe_40_B_20_/ 5 nm Pt	Love wave	433 MHz	−170.4 kHz/mT
Schell 2020 [8]	ST-cut quartz x + 90°	10 nm Cr/300 nm Au/C10 nm Cr	4.5 µm SiO_2_	8 nm Ta/ 200 nm FeCoSiB/ 5 Ta	Love wave	147–148 MHz	2000°/mT (3999 kHz/mT *)
Mishra 2020 [27]	ST-cut quartz x + 90°	100 nm Al	510 nm ZnO	100 nm CoFeB	Love wave	433 MHz	15.53 kHz/mT
Jia 2020 [13]	128°YX LiNbO_3_	300 nm Al	50 nm SiO_2_	50 nm Cr/ 500 nm FeCo (dot array)	-	150 MHz	6 kHz/mT
Xiangli 2018 [4]	ST-cut quartz x + 90°	Ta	SiO_2_	FeCoSiB	Love wave	221.76 MHz	663.98 kHz/mT
Kittman 2018 [5]	ST-cut quartz x + 90°	12 nm Cr/300 nm Au/12 nm Cr	4.5 µm SiO_2_	10 nm Ta/ 200 nm FeCoSiB/ 10 nm Ta	Love wave	147.2 MHz	504°/mT (1008 kHz/mT *)

* The specification in kHz/mT represents an approximation. For data conversion a delay time of 1389 ns has been assumed. This delay time was reported for a similar sensor design as considered here [24].
